# Combined sclerotherapy followed by surgical resection of a large extracranial venous malformation in a 14-month-old child: preoperative workup and technical note

**DOI:** 10.1007/s00381-022-05643-z

**Published:** 2022-08-13

**Authors:** Alexandre Lavé, Hasan Yilmaz, Andrea Rosi, Luca Paun, Gildas Patet, Andrea Bartoli

**Affiliations:** 1 Division of Neurosurgery, Faculty of Medicine, Geneva University Hospitals and University of Geneva, Rue Gabrielle-Perret-Gentil 4, 1205 Geneva, Switzerland; 2 Division of Diagnostic and Interventional Neuroradiology, Faculty of Medicine, Geneva University Hospitals and University of Geneva, Geneva, Switzerland

**Keywords:** Venous malformation, Vascular anomalies, Pediatric cranial surgery, Sclerotherapy

## Abstract

**Introduction:**

Venous malformations affect around 2 per 10,000 newborn and are the most common type of congenital vascular malformation. They are always present at birth and are often misdiagnosed with hemangiomas. Accurate diagnosis and adequate targeted therapy through a multidisciplinary approach is advocated for a successful treatment, considering a combination of modalities in complex cases. We present here the workup and treatment of a venous malformation in a 14 month old child by combining a preoperative sclerotherapy with sodium tetradecyl sulfate (STS) followed by complete surgical excision respecting the calvarium.

Case presentation.

A large right extracranial fronto-parietal venous malformation and scalloping of the underlying calvarium, attached to the pericranium, surgically excised after preoperative sclerotherapy with STS in a 14-month-old child.

**Results:**

The patient had an uneventful postoperative course with complete resection of the lesion, satisfying cosmetic appearance and no complications. Histopathological examination confirmed a venous malformation.

**Conclusion:**

We demonstrated the workup and the rationale of the combined sclerotherapy followed by surgical resection of a large growing extracranial venous malformation. Preoperative direct contrast injection and sclerotherapy allowed to rule out significative extracranial to intracranial venous inflow and reduce blood loss for the surgical procedure, respectively. Complete removal of the malformation minimized the impact on future growth of the calvarium.

## Introduction

Venous malformations (VMs) have been described by the Mulliken and Glowacki classification of vascular anomalies in 1982 [1]. They are classified as low-flow vascular malformations in opposition to high flow lesions such as arteriovenous malformation and arteriovenous fistula. These malformations are well distinguished from vascular tumors (most often hemangiomas) based on endothelial characteristics, as VMs are composed of normal endothelial-lined vascular spaces without hypercellularity, whereas hemangiomas are characterized by several histological abnormalities, including hyperplasia. The Mulliken and Glowacki classification has been revised since its creation, and the ISSVA (International Society for the Study of Vascular Anomalies) classification of vascular anomalies [2] is now used to define vascular anomalies (as shown in Table 1).

**Table 1:** ISSVA classification for vascular anomalies 2014, revised in 2018. Adapted from https://www.issva.org/classification

**Vascular tumors**	**Vascular malformations**			
**Benign**	**Simple**	**Combined**	**Of major named vessels**	**Associated with other anomalies**
	Capillary malformations	CVM (capillary-venous malformation)	Affect: - lymphatics - veins - arteries Anomalies of: -origin -course -number -length -diameter (aplasia, hypoplasia, stenosis, ectasia/aneurysm) -valves -communication (AVF) -persistence (of embryonal vessel)	Klippel-Trenaunay syndrome Parkes-Weber syndrome Servelle-Martorell syndrome Sturge-Weber syndrome **Limb CM + congenital non-progressive limb overgrowth** **Maffucci syndrome** **Macrocephaly—CM (M-CM/MCAP)** **Microcephaly-CM (MICCAP)** **CLOVES syndrome** **Proteus syndrome** Bannayan-Riley-Ruvalcaba syndrome CLAPO syndrome
		CLM (capillary-lymphatic malformation)		
Locally aggressive or borderline	Lymphatic malformations	LVM (lymphatic-venous malformation)		
		CLVM (capillary-lymphatic-venous malformation)		
Malignant	Venous malformations	CAVM (capillary-arteriovenous malformation)		
	Arteriovenous malformations	CLAVM (capillary-lymphatic-arteriovenous malformation)		
	Arteriovenous fistulas	Others		

The revised version of 2018 continues to divide vascular anomalies into vascular tumors and vascular malformations and classifies vascular malformations in simple or combined (if two or more vascular malformations are found in one lesion) rather than high or low flow. Associated syndromes (Klippel-Trenaunay, blue-rubber bleb nevus, familial cutaneomucosal venous malformation, Maffucci’s) and specific mutations (TIE2 mutation) have been also included in the classification.

VMs are always present at birth by definition, thus have to be considered congenital lesions, and grow in parallel with the child's development [3, 4], with a peak from infancy to puberty. VMs may be very small initially and thus be identified only later in childhood or teenagehood [5, 6]. Both sexes are equally affected with a reported incidence of 1–2 per 10,000 births and a prevalence of 1% [5, 6].

Clinically, these lesions are superficial, soft, without pulsations, thrill, or increase of temperature relative to adjacent skin, in contrast to arteriovenous malformations. The size of the VM can increase with activity, Valsalva, or supine position [5, 7, 8]. Classical appearance changes from light to dark-blue lesions.

VMs can sometimes be painful, especially in cases of facial VMs, if the temporal muscle is affected (patients can complain of migraine). Patients could also present with swelling and pain due to thrombosis of a portion of the lesion [5].

Diagnostic workup should start first with ultrasonography (US) to identify the classic hypoechoic, heterogeneous, and compressible appearance of VMs. Duplex is then used to show the low flow and identify the feeding vessels and intralesional thrombosis. Duplex ultrasound is also the best modality to differentiate hemangioma (high flow on duplex) from VMs (low flow). This discrimination is important because in case of misdiagnosis, inappropriate beta-blocker therapy may be proposed (9). MRI T1- and T2-weighted sequences are considered the gold standard to assess VMs [10, 11]. On fat-saturated T2-weighted MR, VMs appear hyperintense, heterogeneous with lobulated margins. Phleboliths are sometimes visible inside the lesion. In selected cases, contrast venogram, CT scan, or even scintigraphy could be useful [5].

Treatment options include medications, surgery, sclerotherapy, cryoablation, and laser photocoagulation; the decision for treatment should be ideally done after interdisciplinary discussion. Small well-localized VMs are often treated successfully with a single modality (surgery, sclerotherapy), whereas larger venous malformations may require a combination of technique [3–5, 9].

Compressive therapy is a simple and efficient option mostly for VMs of the extremities to reduce pain and thrombosis; targeted medical treatment consists of low-dose aspirin, anti-inflammatory drugs, or low-molecular-weight heparin in cases of pain associated with thrombophlebitis [12, 13].

The modality of choice for the treatment of VMs is considered to be sclerotherapy, which can be curative, or used prior to surgery as a neo adjuvant solution, mostly to induce thrombosis inside the lesion to facilitate excision [12]. Many different sclerosants are available, including alcohols, detergents, antitumor agents, and doxycycline. Alcoholic agents are considered to be the most effective, with a very low recurrence rate [12], but also associated with an increased risk of lesions for adjacent skin, nerves, and soft tissues.

Surgery alone is sometimes preferred to sclerotherapy, especially when the venous malformation is small and does not involve vital structures; the use of sclerotherapy as an neoadjuvant therapy is often advocated mostly to reduce intraoperative blood loss, to decrease the volume of the malformation to be resected, and to improve visualization of vital structures.

## Case presentation

We present here the case of a 14-month-old child, without any relevant past medical history, with a large right fronto-parietal soft tissue mass, more prominent in the supine position, and which gradually increased in size with the child growth. Final size was estimated around 7 cm in maximal diameter. The perinatal history was unremarkable, the delivery uneventful, and no previous trauma was recorded. The child’s weight at the time of presentation at our clinic was around 11 kg. The child did not show any particular complaint in relation to this lesion.

**Fig. 1:**
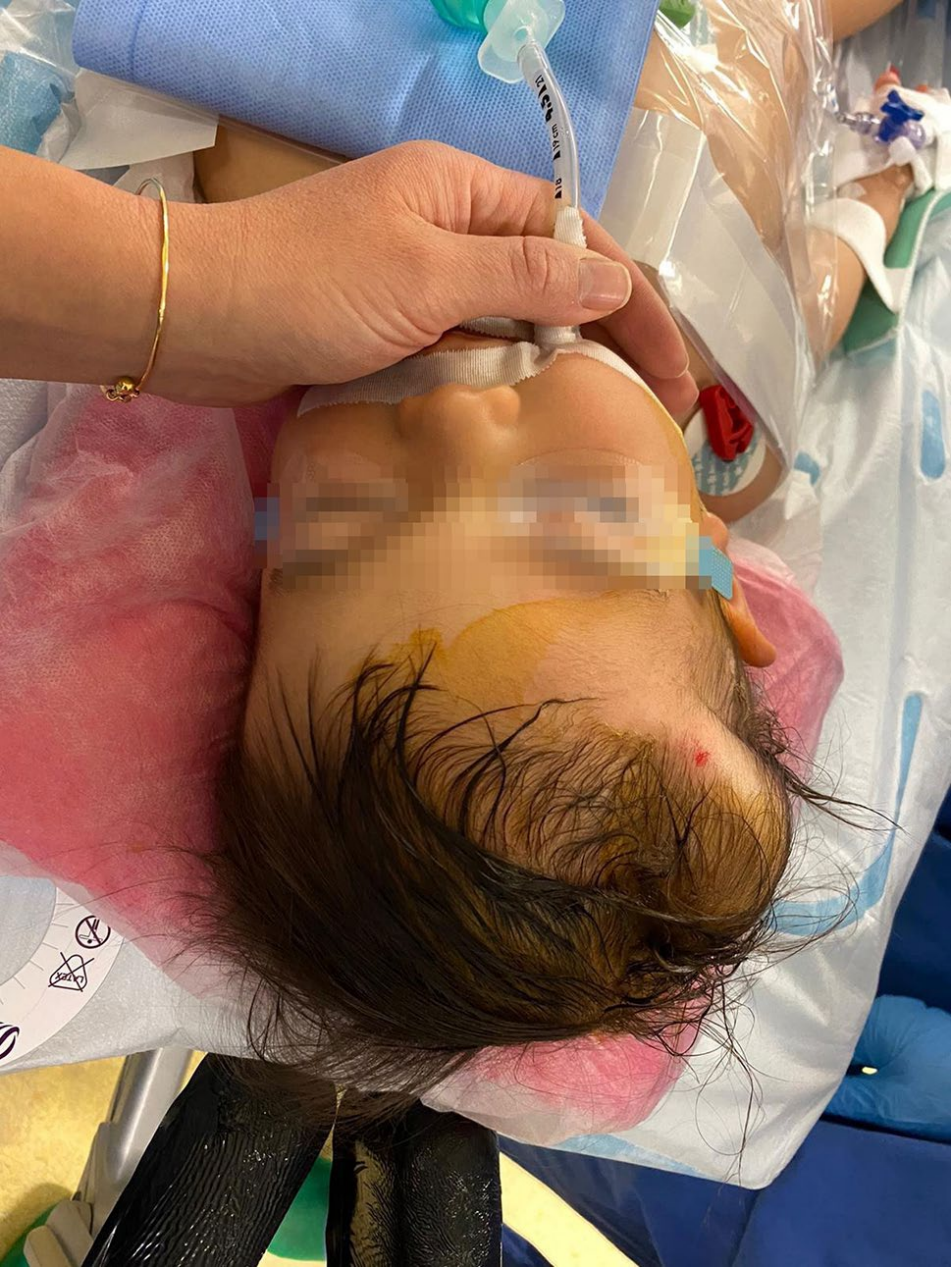
Inspection of the 7 × 3 cm subgaleal venous malformation prior to the surgery. Note that the skin mark in the frontal part was due to Trombovar injection; the overlying skin is intact

Clinical examination showed a soft lesion, not mobile to deep structures of the scalp. Recumbent position showed an enlargement of the lesion. The skin was intact, and the rest of the inspection and palpation of the cranial vault were normal (as shown in Fig. 1). No neurological deficits were observed.

**Fig. 2:**
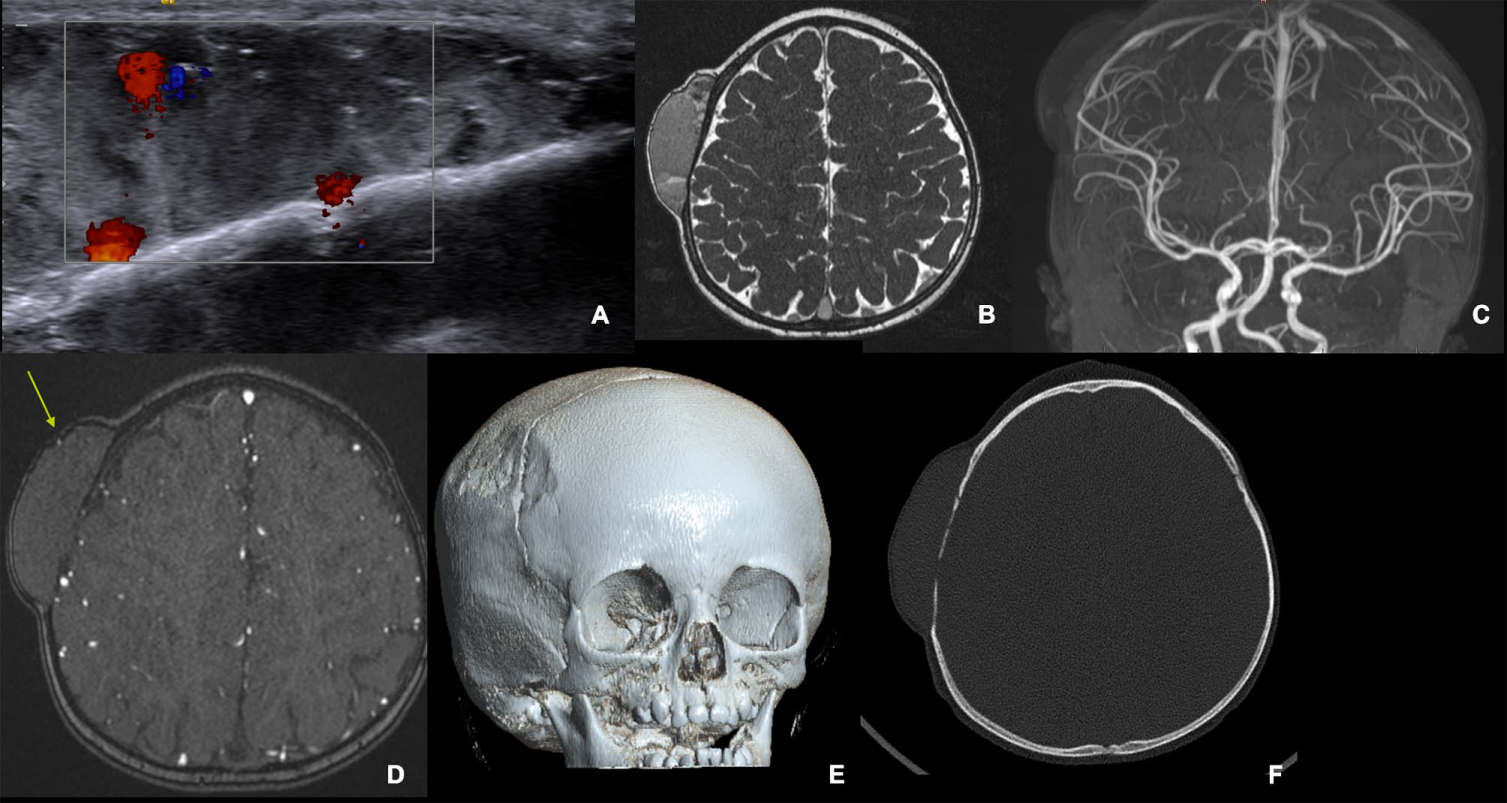
Pre-operative imaging workup. **A** Doppler ultrasonography showing the low-flow heterogeneous content, without intradural venous drainage through the bone lacunae. **B** Brain 3D T2 MRI sequences in the axial and coronal planes with heterogeneous signal, sharp boundaries, and with internal septations inside the mass. **C**, **D** The MRI TOF sequences did not show any communication with the intracranial vessels. The yellow arrow shows the frontal branch of the superficial temporal artery (STA). **E**, **F** Scalloping of the calvaria bones is evident under the mass on the CT scan

The presurgical work up began with a Doppler ultrasonography which showed a deep lenticular soft tissue formation, purely extracranial, with heterogeneous content. There was no sign of arterial feeding by large-caliber intracranial or extracranial arteries. The Doppler analysis showed a low flow, without any visible venous drainage at the cortical level through the bony lacunae (as shown in Fig. 2).

Scalloping and lacunae of the calvarium underlying the lesion were visible on the CT scan of the head (as shown in Fig. 2).

Cerebral magnetic resonance imaging (MRI) revealed large vascular spaces, with minimal intracranial extension abutting the dural surface through bony lacunae. No extra to intracranial shunts were demonstrated on TOF sequences. Heterogeneous signal, sharp boundaries, and serpiginous content with a low flow were highly suggestive of a vascular lesion with venous characteristics.

After a multidisciplinary meeting, including neurosurgeons, interventional neuroradiologists, dermatologists, angiologists, and pediatric surgeons, surgery was offered to parents because of the significant cosmetic discomfort, the tendency of the mass to grow, and the scalloping of the calvarium.

We decided to perform the procedure in a hybrid operating room, with preoperative direct contrast injection and sodium tetradecyl sulfate (STS) sclerotherapy. The aim of the direct contrast injection followed by sclerotherapy was to rule out any extra to intracranial venous shunt and to reduce blood loss respectively, prior to surgery.

### Angiographic procedure

The patient is in supine position; antibiotic prophylaxis with intravenous cefazolin 25 mg/kg is given prior to the endovascular procedure.

**Fig. 3:**
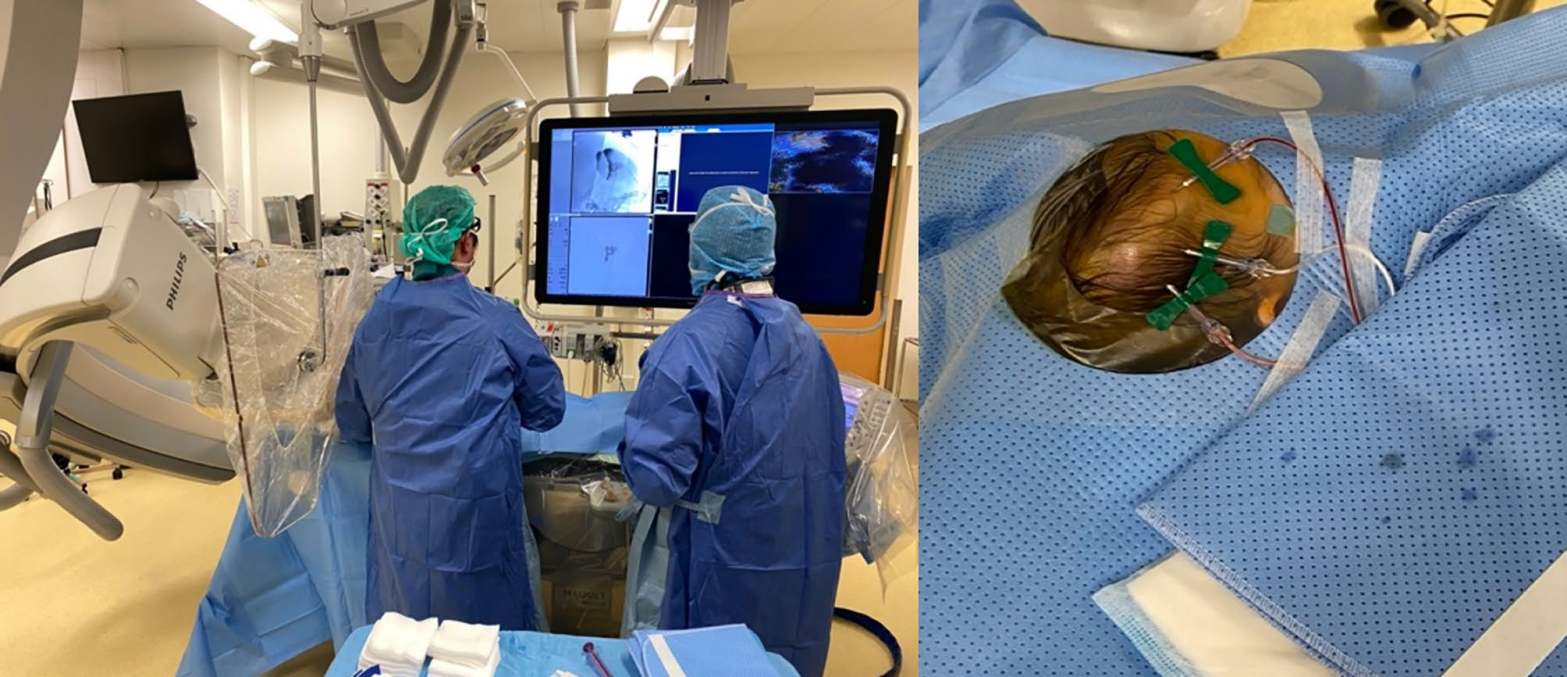
Pre-surgical percutaneous sclerotherapy procedure in the hybrid operating room/angio-suite. Multiple 21-gauge butterfly needles were placed in the different compartments of the venous malformation

The angiographic procedure began with a direct injection of contrast (30 mL of Iopamiro 300 mg/mL) into the lesion (as shown in Fig. 3). It confirmed the presence of a right fronto-parietal vascular multiloculated malformation of about 7 × 3 cm.

**Fig. 4:**
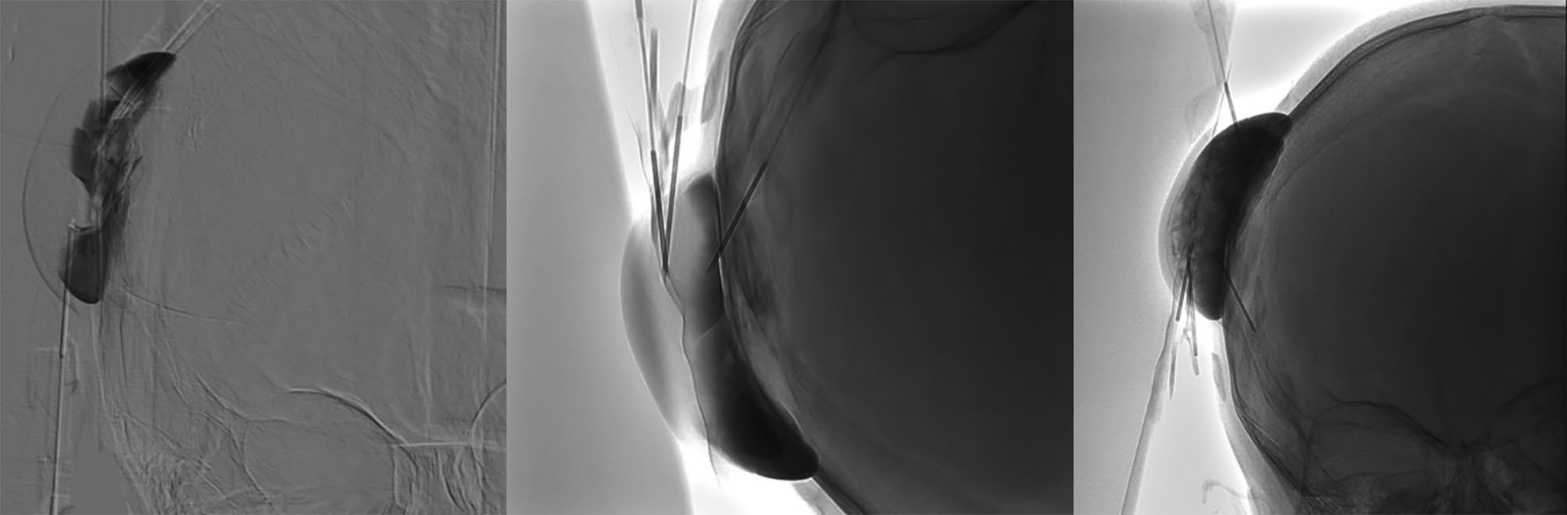
Sequential injection of trombovar 1% during the sclerotherapy (from left to right) showing the complete opacification of the mass without any signs of diffusion into the dural veins

The anterior compartment of the malformation was connected to 2 small dural veins (one running cranially into to the superior sagittal sinus and the other to the basal dura) and one small frontal cortical vein. After ruling out any extra to intracranial venous shunt, sclerotherapy was slowly performed via direct puncture of the lesion using 4 21-gauge butterfly needles in the different compartments. We injected 4 mL of sodium tetradecyl sulfate foam (Trombovar® 1%) mixed with contrast and air (in proportion of 1:1:1). No diffusion to the previously described veins was seen under fluoroscopy during the procedure (as shown in Fig. 4).

### Surgical procedure

**Fig. 5:**
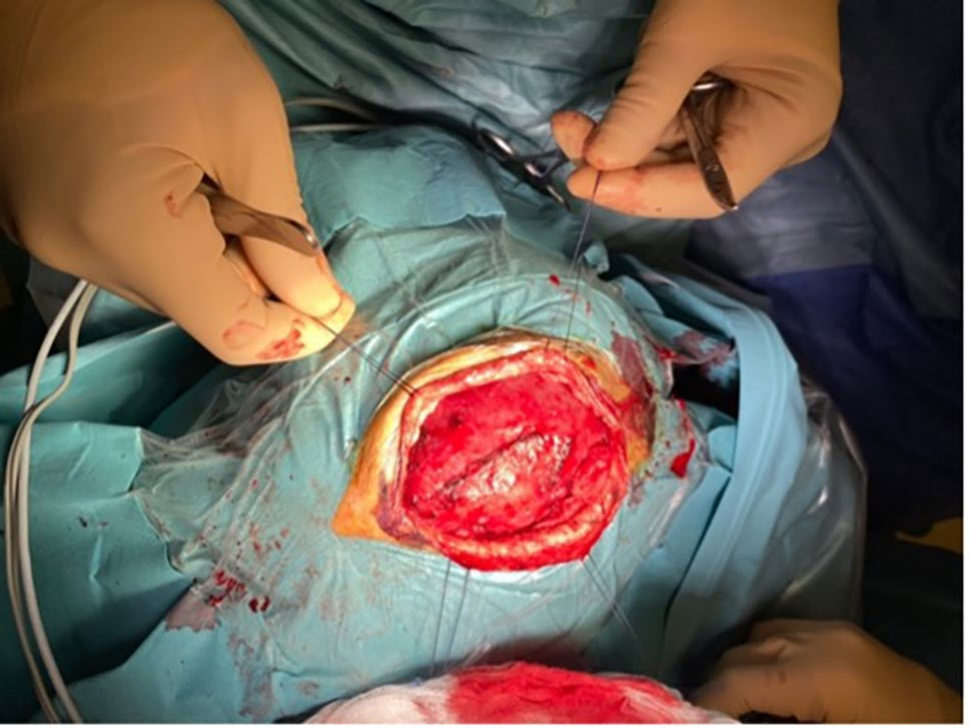
Complete exposure of the margins of the venous malformation between the galea and the subcutaneous plane

We marked a linear incision line, limited by the hair line. A right fronto-parietal arciform incision with a cold scalpel was done followed by a monopolar dissection to expose the galea. The lesion was lying within the subaponeurotic plane between the galea and the pericranium. Subsequently, dissection with Metzenbaum scissors was performed following a plane between the subcutaneous layer and the galea (as shown in Fig. 5). Keeping an extracapsular dissection, we detached the vascular malformation with the pericranium from the underlying bone.

**Fig. 6:**
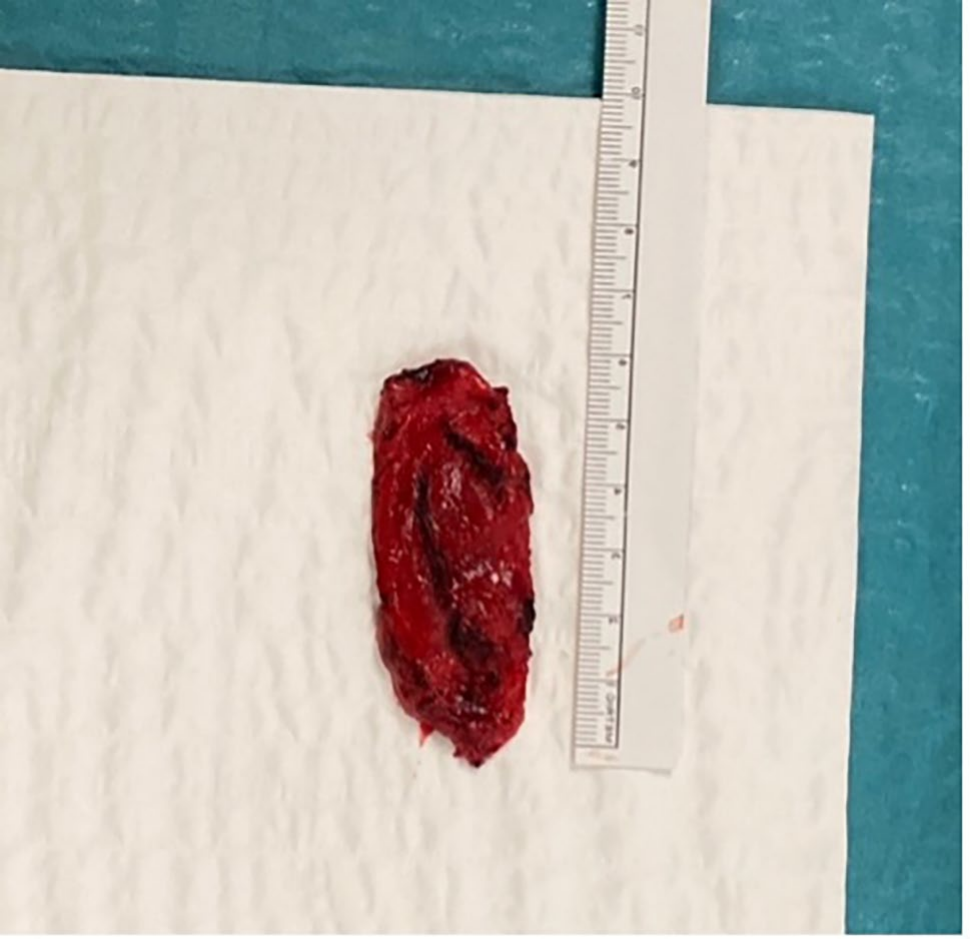
En-Bloc resection of the venous malformation

We observed an emptying of the vascular malformation during its dissection and we finally achieved an en-bloc resection (as shown in Fig. 6).

**Fig. 7:**
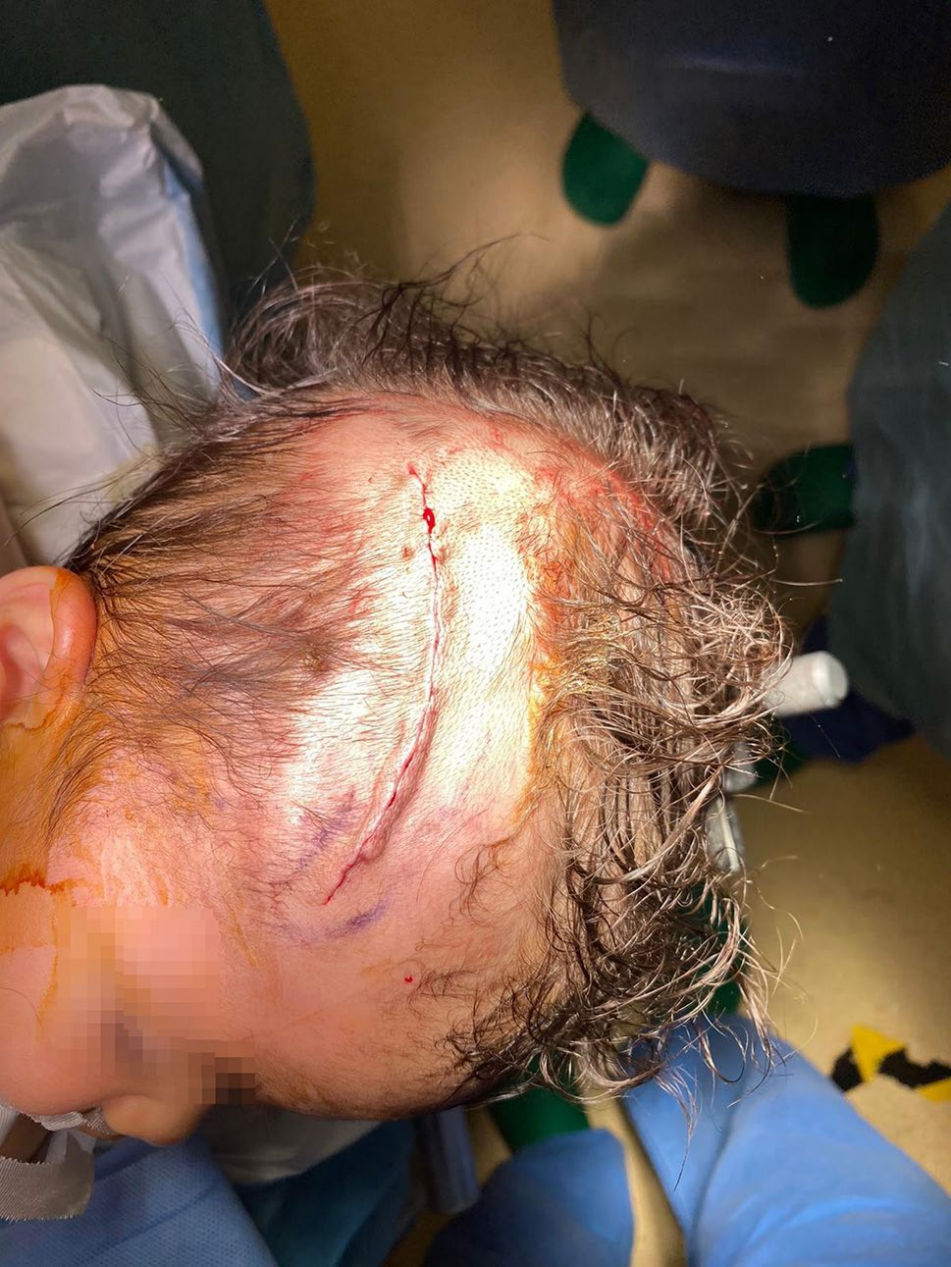
Intradermal suture allowed an excellent esthetic result, without excess of cutaneous tissue

Bone wax, Spongostan^®^ (Ethicon™), and Evicel^®^ (Ethicon™) were used for hemostasis. The deep plane and subcutaneous plane were closed with Vicryl^®^ 3.0. An intradermal suture of Monocryl^®^ 3.0 was used to close the skin and a pressure dressing was applied for 3 days (as shown in Fig. 7). Total hematic loss of the entire procedure was estimated between 50 and 100 cc.

### Postoperative follow-up

Surgery was performed without perioperative complications. Pain was controlled by a combination of NSAIDs and paracetamol from post-operative day (POD) 1. Immediate postoperative follow-up was unremarkable and patient was discharged at POD 4.

## Conclusion

To our knowledge, this is the first description of the successful treatment of a cranial venous malformation threatening calvarium development in a pediatric case by combining sclerotherapy to surgical excision. Pre-operative sclerotherapy allowed easier manipulation, decreased the per operative blood loss, and shortened surgical procedure and therefore the length of general anesthesia. Finally, by a direct injection of contrast into the malformation at the beginning of the procedure, we were able to rule out any significative extra to intracranial venous shunt.

Management of venous malformations requires a multidisciplinary approach from the diagnostic workup to the treatment. It should involve interventional radiologists or neuroradiologists, surgeons, dermatologists, and angiologist in order to maximize the success rate while avoiding iatrogenic complications.

## Data Availability

Publicly available datasets were used in this study. Authors confirmed that the data supporting the study are available within the article in the References section.

## Abbreviations

VMs: Venous malformations
STS: Sodium tetradecyl sulfate
MRI: Magnetic resonance imaging
POD: Post-operative day
